# A Rare Case of Disseminated *Mycobacterium avium-intracellulare* Presenting as Proctitis

**DOI:** 10.1155/2019/8129597

**Published:** 2019-12-03

**Authors:** Chukwunonso Chime, Peter Bhandari, Masooma Niazi, Harish Patel

**Affiliations:** ^1^Division of Gastroenterology, BronxCare Hospital Center a Clinical Affiliate of Mt Sinai Health Systems and Academic Affiliate of Icahn School of Medicine, Bronx, NY 10457, USA; ^2^Department of Medicine, BronxCare Hospital Center a Clinical Affiliate of Mt Sinai Health Systems and Academic Affiliate of Icahn School of Medicine, Bronx, NY 10457, USA; ^3^Department of Pathology, BronxCare Hospital Center a Clinical Affiliate of Mt Sinai Health Systems and Academic Affiliate of Icahn School of Medicine, Bronx, NY 10457, USA

## Abstract

*Mycobacterium avium intracellulare* (MAI) infections are common in *Human Immuno-deficiency Virus* (HIV) positive patients. MAI infection can have localized or disseminated presentation, patients with low CD4 count presenting with disseminated infection. Fever, abdominal pain, diarrhea, and weight loss are generally the presenting symptoms of disseminated MAI. We present a rare case of a patient with HIV and low CD4 count presenting with proctitis as manifestation of disseminated MAI infection. A 25 year-old-man with HIV came to the emergency room (ER) with complaints of intermittent rectal bleeding for two months. His CD4 count was less than 20 cells/*µ*L. He was MSM (men having sex with men) and has receptive anal intercourse with men. His stool work-up was unrevealing for infectious etiology. Swabs for gonorrhea and chlamydia were negative. Colonoscopy revealed erythematous, congested, friable rectal mucosa with two superficial ulcers. Biopsies of the ulcer were positive for acid fast staining bacteria and the culture grew MAI. His blood culture was negative for growth of acid-fast bacteria (AFB). However, liver biopsy performed for elevated alkaline phosphatase of 958 units/L revealed noncaseating granuloma. Gastro-duodenoscopy with duodenal biopsy did not reveal any mucosal abnormality. He was managed as with disseminated MAI infection using clarithromycin, ethambutol, and rifabutin in addition to HAART therapy. Interval Colonoscopy in 20 months showed resolution of rectal ulcer. The gut is often involved in patients with disseminated MAI infection, with the duodenum being the most common site. MAI infection should be suspected as possible etiology for proctitis in HIV positive patient with low CD4 count, as proctitis, though infrequent can be the sole presentation for disseminated MAI infection in patients with HIV and low CD4 count.

## 1. Introduction

The association between *Mycobacterium avium intracellulare* (MAI) and acquired immune deficiency syndrome (AIDS) was identified in the 1980's [1]. Immune compromise status in general is a risk factor for acquiring MAI infection. The epidemic of MAI is concurrent to the AIDS epidemic [2] with a low CD4 count being a risk factor for acquiring MAI infection [3]. With advent of Highly Active Anti-Retroviral Therapy (HAART) there has been a control of MAI epidemic, however, nonadherence to HAART can decrease the CD4 count and hence increase susceptibility to MAI infection [4]. The occurrence of MAI infection is relatively late in the course of the natural history of Human Immuno-deficiency Virus (HIV) infection [5] and with improvement in life expectancy in the era of HAART, there can be an increased frequency of MAI infections [5, 6].

MAI infection can present as a localized disease or a disseminated presentation, patients with AIDS being more prone to disseminated infection. Blood stream infection, lymph nodes, and liver infections are common in patients with disseminated MAI. Gastrointestinal manifestations are also common in patients with systemic MAI infections, the duodenum being the most common organ to be involved [7]. We present a case of a young male with AIDS presenting with proctitis and diagnosis of the disseminated MAI, successfully treated with triple combination therapy.

## 2. Case Presentation

A 25-year-old male presented to the emergency room (ER) with complaints of rectal bleeding, described as bright red blood on formed stool, for 2 months. His medical co-morbid condition includes a 7-year history of Human Immuno Deficiency Virus (HIV) infection, nonadherence to Highly Active Retroviral Therapy (HAART) with a CD4 count <20 cells/*µ*L and HIV viral load of 151,257 copies/mL. He is MSM (men having sex with men) and has receptive anal intercourse with men. He reported remote treatment for Genital Herpes. In the ER, his vitals were within normal limits and physical examination was unremarkable except for minimal right upper quadrant tenderness upon palpation and numerous tender perirectal ulcerations. Rectal examination revealed normal sphincter tone with empty but tender rectal vault. There was no peripheral lymphadenopathy.

**Figure 1:**
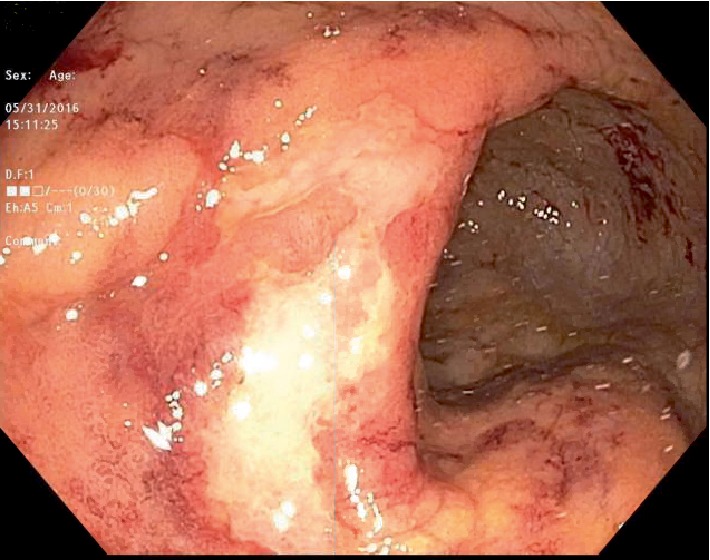
Rectal mucosal with ulcerations.

**Figure 2:**
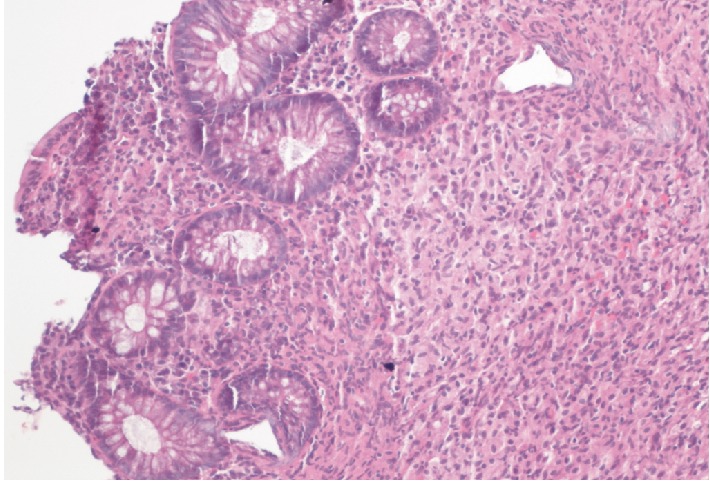
Rectal biopsy showing diffuse expansion of lamina propria with infiltration by lymphohistiocytic cells. (*H* and *E*, magnification ×200).

**Figure 3:**
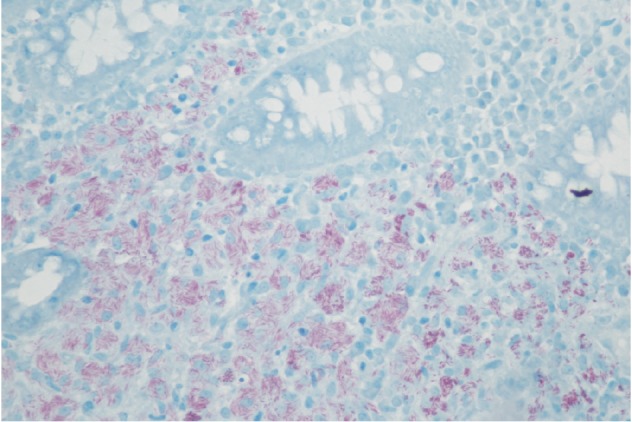
Rectal biopsy: acid fast staining showing sheets of macrophages packed with bundles of acid-fast bacilli, characteristic of *Mycobacterium avium intracellulare*. (Acid fast stain, magnification ×400).

Laboratory investigations showed haemoglobin of 10.4 g/dL, leukocyte count of 3.1 K cells/*µ*L, platelet count of 339 K cells/*µ*L, aminotransferase levels 3X ULN, total bilirubin levels of 7.0 mg/dL with conjugated bilirubin of 5.2 mg/dL, serum lactate dehydrogenase of 327 unit/L, serum gamma glutamyl transferase of 414 units/L, and alkaline phosphatase of 958 unit/L. Stool work up was negative for *Cryptosporidium*, *Cryptococcal* antigen, *Clostridium difficile*, *Cyclospora*, *Microsporidium*, and *Isospora*. No Ova and parasites isolated from stool studies. Blood cultures for regular pathogens acid-fast bacteria (AFB) were negative. Patient was admitted and colonoscopy performed to further identify the cause of rectal bleeding revealed diffusely erythematous, congested, friable rectal mucosa with two superficial ulcers (Figure 1). Biopsies were taken of the rectal mucosa as well as normal appearing proximal colon and terminal ileum. Rectal swabs were negative for chlamydia and gonorrhea. Pathology for rectal biopsy revealed AFB positive organism (Figures 2 and 3) and culture revealed growth of *Mycobacterium avium-intracellulare (MAI)* organisms. During this clinical course, he had an unremarkable upper endoscopy and was started on clarithromycin 500 mg BID, ethambutol 1200 mg QD, and rifabutin 300 mg QD in addition to HAART therapy.

**Figure 4:**
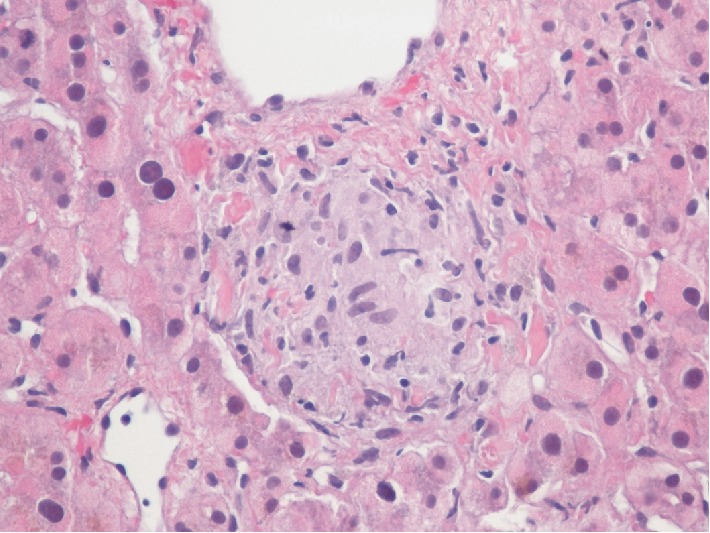
Liver biopsy with nonnecrotizing granuloma. Composed of lymphohistiocytic collections. (*H* and *E*, magnification ×400).

**Figure 5:**
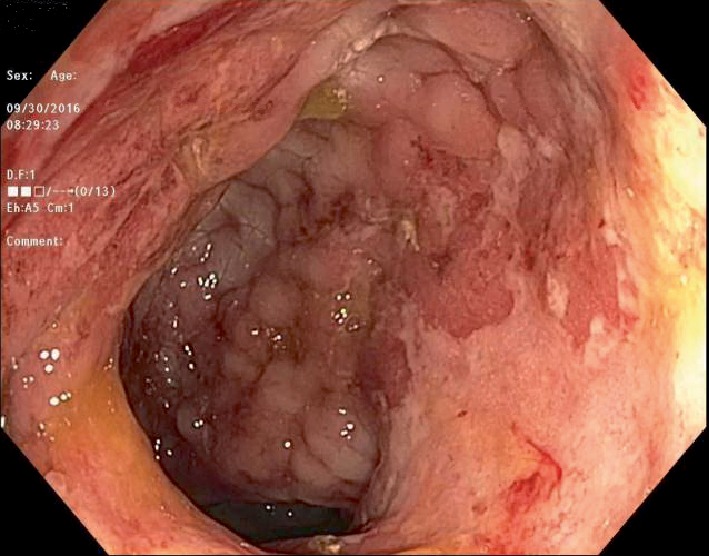
Friable rectal mucosa with edema (2 months after initiation of MAI treatment).

Clinical presentation was highly suspected for disseminated MAI infection. Given negative blood culture, we further pursued evaluation of elevated alkaline phosphatase and performed liver biopsy. Pathology was reported to have necrotizing granulomas composed of lymphohistiocytic collections (Figure 4), highly suggestive of MAI infection. This confirmed our diagnosis of disseminated MAI. Patient was on and off the recommended regimen, flexible sigmoidoscopy after a short course of interrupted treatment again demonstrated similar initial findings in the rectum (Figure 5) but a follow up colonoscopy 20 months after diagnosis showed healing of rectal mucosa with negative biopsies for MAC. There was also a decreasing trend of alkaline phosphatase while on treatment, with last reported value of 218 unit/L.

## 3. Discussion

Nontuberculous Mycobacteria (NTM) are environmental organisms that are opportunistic in nature and under favorable conditions, cause a wide range of diseases ranging from pulmonary to disseminated infections, with the former being the most common [8]. Immune suppression is the typical predisposing factor and includes patients with HIV/AIDS, using immunosuppressive therapy and undergoing solid organ or haematologic transplant [9]. A CD4 count below 50 cells/*µ*L presents the highest risk for NTM infection and there has been suggestion of a genetic predisposition determined by human leukocyte antigen (HLA) class [10]. In the context of immune suppression, the vast majority of NTM cases are still *Mycobacterium avium complex* (MAC) but several factors lead to increased recognition of other species like *M. fortuitum*, *M. abscessus*, *M. chelonae*, and *M. haemophilium*, these include host factors and improved laboratory diagnostics [9]. In the United States, Tuberculosis (TB) has been overtaken by NTM as the predominant cause of mycobacterial lung disease [11].

Proctitis can be from infectious or noninfectious etiology. In MSM (men having sex with men) population, proctitis presents as sexually transmitted disease with gonorrhea being the most common infection [12]. MAI related proctitis is an uncommon presentation and it is not considered to be a sexually transmitted disease. Localized and disseminated disease are the two presentations of MAC in patients infected with HIV with the latter declining with increasing use of HAART [13]. Disseminated MAC present with malabsorption, rectal bleeding and loose stool when the gastrointestinal tract is involved [14], with the duodenum being the most commonly involved location in a case series [7]. In patients with advanced AIDS, MAI is one of the most common etiology of hepatic opportunistic infection [15]. It has a varied clinical manifestation ranging from granulomatous liver disease to presentation of a liver mass [16]. The increase in alkaline phosphatase in our patient appears to arise from MAI induced granulomatous liver disease and with anti-microbial management for MAI, there was an interval decrease in alkaline phosphatase levels. Liver involvement is a sign of disseminated MAI infection. Once diagnosis of MAC is derived from liver biopsy, endoscopic work-up is usually not warranted to start anti-microbial treatment. Hence, there is literature to opine on the rates of rectal infection in conjunction with liver MAI involvement.

In general, cultures are the mainstay of diagnosis of infection with MAI. Blood cultures are preferred in disseminated cases as they are highly sensitive [13]. There is a role for stool culture in early diagnosis of patients with disseminated disease, especially in cases with confirmed gastrointestinal involvement, with sensitivity and specificity as high as 86% and 99% respectively [7, 17]. In our patient, stool culture was largely negative and did not reveal any opportunistic pathogen. Upper endoscopy and colonoscopy can aid in visual identification of any abnormalities in the mucosa and result in tissue specimens that can be stained and directly observed for acid fast bacilli present in the setting of noncaseating granuloma as was the case in our index patient. In patients with MAI colitis, erythema, friability, and ulcerations are commonly seen endoscopic findings [14], these were also noted in our patient and shown in Figures 1 and 5 above. A case of MAI presenting with severe gastrointestinal bleeding in a non HIV patient, diagnosed with colonoscopy with biopsy specimens staining positive for acid fast organisms, was described by Nguyen et al. [18].

In a nonHIV patient with extrapulmonary MAI, given that lack of consensus on the duration of treatment, a minimum duration of 6 months is usually adopted, meanwhile the pulmonary cases are tailored towards treatment for 12 months after sputum cultures have remained negative [19]. The main stay of treatment for MAI is with dual therapy comprising of ethambutol and macrolides, especially in patient with AIDS [13]. Some special circumstances that warrant a third agent include elevated mycobacterial burden, lack of antiretroviral therapy and patients with advanced immune compromise [20]. The preferred third agent is usually Rifabutin but other choices could be amikacin or fluroquinolones like moxifloxacin and levofloxacin, these combinations are typically used for at least 12 months, preferably for minimum of 6 months after sustained CD4 count above 100 cells/*µ*L [20].

Our patient was started on triple therapy, issues were encountered during his treatment course given his poor adherence. But after 20 months of therapy, he had clinical and endoscopic cure of his MAI proctitis. He is currently on treatment for the MAI, concurrent with his HAART given that his CD4 count is still not at target.

## 4. Conclusion

Disseminated MAI with gastrointestinal involvement should always be considered as a cause of lower gastrointestinal bleeding, in patients with immune compromise. The findings, including friability, and ulcerations, are nonspecific and can be seen in other clinical entities that include but are not limited to inflammatory bowel disease and cytomegalovirus colitis, both of which are commonly seen with immune deficiency. Biopsy with special stains helps to identify MAI as the causative organism. Treatment will depend on immune status and location, with extra pulmonary cases like our index case requiring at least 12 months of treatment. Given the correlation between this opportunistic pathogen and low CD4 count in the HIV patients, concurrent treatment with HAART is also warranted.
